# The Cultural Politics of ‘Implementation Science’

**DOI:** 10.1007/s10912-020-09607-9

**Published:** 2020-01-21

**Authors:** Richard Boulton, Jane Sandall, Nick Sevdalis

**Affiliations:** 1 St George’s University of London, London, UK; 2 Kingston University, Kingston upon Thames, UK; 3 King’s College London, London, UK

**Keywords:** Implementation science, Interdisciplinarity, Qualitative vs quantitative

## Abstract

Despite the growing profile of ‘implementation science’, its status as a field of study remains ambiguous. Implementation science originates in the evidence-based movement and attempts to broaden the scope of evidence-based medicine to improve ‘clinical effectiveness’ and close the ‘implementation gap’. To achieve this agenda, implementation science draws on methodologies from the social sciences to emphasise coherence between qualitative and quantitative approaches. In so doing, we ask if this is at the expense of ignoring the dominating tendencies of the evidence-based movement and consider if some of the methodologies being drawn on should be considered irreconcilable with evidence-based methodologies.

## Introduction

In 2006 a new journal, *Implementation Science*, was founded to focus on bridging the ‘implementation gap’ between research ‘evidence’ and its adoption into practice. The founding principles of the journal defined its scope as:

…the scientific study of methods to promote the systematic uptake of research findings and other evidence-based practices into routine practice, and, hence, to improve the quality and effectiveness of health services and care. (Eccles and Mittman 2006)

Since then the journal has been developed as the centrepiece of a whole field aiming to formalise the implementation of ‘evidence-based’ research findings into practice. Accompanied by a UK and US political agenda, implementation science’s purpose is to ‘improve the quality and effectiveness of health services and care’. Through implementation science, the social sciences appear to be gaining more ground in evidence-based healthcare. Qualitative methodologies, which were once low on the agenda of healthcare research, now have a privileged position through the development of implementation science. This is exemplified in leading implementation science frameworks such as the Consolidated Framework for Implementation Research (CFIR); Promoting Action on Research Implementation in Health Services (PARIHS); and the Theoretical Domains Framework (TDF) (see Table 1). However, what has been less explored in the field is how implementation science (and frameworks such as these) effectively broadens the scope of evidence-based medicine and may reconfigure the balance between methodologies and epistemologies in healthcare. This balance is important to assess as it informs how ‘quality and effectiveness’ is defined, and whose benefit it serves. Rather than evidence-based medicine being confined to the effectiveness and efficiency of medical treatments, now many previously unobserved social practices and behaviours have come under the scope of evidence-based methodologies.

**Table 1:** Some leading models of Implementation Science discussed in the text. Their Rationale and Method have been summerised using their own wording in order to demonstrate their epistemological and methodological commitments

1**) Consolidated Framework for Implementation Research (CFIR)** (Damschroder et al. 2009) **Rationale:** Designed to standardise implementation theory, "each [theory] is missing one or more key constructs included in other theories. [CFIR is] a comprehensive framework that consolidates constructs found in the broad array of published theories" (p2). CFIR synthesises implementation models into 5 domains. **Method**: Greenhalgh et al. 2004 was [their] "starting point for the CFIR. [They] used a snowball sampling approach to identify new articles through colleagues [...] and theories that cited Greenhalgh *et al*.'s synthesis, or that have been used in multiple published studies in health services research (e.g. PARIHS)" (p2).	
2) **Diffusion of Innovations in Service Organisations** (Greenhalgh et al. 2004) **Rationale:** Aims to offer a way "to define and measure the diffusion of innovations in organisations" (p581). Provides a "basis across a wide range of literature" to explain the "spread" of ways in which innovation can be understood. **Method:** "To explore this large and heterogeneous literature, [they] developed a new technique, which [they] called *meta-narrative review*" (p583). Devised as a way to carry out a type of meta-synthesis of qualitative as well as quantitative literature.	
3) **Normalisation Process Theory** (NPT) (May & Finch, 2009) **Rationale:** "Puts forwards a theory how and why things become, or don't become, routine and normal components of everyday work" (p535). The model "provides a set of sociological tools to understand and explain the social processes that frame the implementation of material practices" (p540). Proposes a theory of 5 components. **Method**: Revised over two iterations "from secondary analyses of multiple qualitative studies in health care settings". The most recent second iteration "focuses on general processes by which material practices come to be embedded in their social contexts [...]using as exemplars ethnographic and other studies of the development implementation, and evaluation of a tele-dermatology service" (p539).	
4) **The Promoting Action on Research Implementation in Health Services** (PARIHS) (Rycroft-Malone, 2004) **Rationale**: Conceptual framework developed to promote action. Depicts "successful research implementation as a function of the relationships among [3 elements]: evidence, context, and facilitation." (p298) **Method:** The concepts derive from "theoretical and retrospective analysis of 4 studies that had been undertaken by the Royal College of Nurses Institute" (p298). These were programmes run mostly by nurses to "help improve the quality of their care by setting clinical standards, introducing audit and quality improvement, and in changing patient services in several community hospitals in one health authority." (Kitson et al. p150)	
5) **Theoretical Domains Framework** (TDF) (Cane et al. 2012) **Rationale:** A framework developed "to simplify and integrate a plethora of behaviour change theories and make theory more accessible to, and usable by, other disciplines" (p2). Sorts organisational behaviour originally into 12 domains and 14 domains in the refined model. **Method**: Expert selection of "33 theories and 128 key theoretical constructs related to behaviour change and synthesis them into a single framework" (p2).	

The appropriation of qualitative methodologies from the social sciences into applied healthcare research and the relative youth of the field highlight the need to appraise the use of theory and methodology independent of evidence-based methodologies (Foy et al. 2015). In particular, there is a need to consider the idea that theories and frameworks are not just a means to explain a simplified system. Users should have some appreciation of the way a theory conceptualises and represents reality, how theory is a simplification of reality and therefore, be aware of the assumptions a theory is making during its application. Similarly, methodology is not a simple application of method or a demonstration of a set of methods in the style of a tool kit. Methodology is also shaped by appreciations of the ways realities are conceived, and how those realities are accessed through methods. We argue that the danger of not appraising evidence-based methodologies against the broader social sciences, implementation science is attempting to draw from means that practitioners may not be fully aware of in the healthcare systems they are attempting to implement and in whose interests recalibrations of healthcare systems or knowledges may lie. Therefore, we analyse how implementation has emerged as a research agenda, and which social sciences and methods have been enveloped or avoided.

We argue that the general approach of theory and method in implementation science is caught between interpretivist and positivist methodologies, which obscures criticism. This is the result of implementation science’s attempting to defend itself as a field of study amongst other heavily populated social sciences whilst still upholding its main impetus as an offshoot of the evidence-based healthcare movement. Implementation research tied too implicitly to the empirical sciences obscures some of the rich critical methodological traditions implementation science is now starting to draw from. Any attempts to make the field compatible are inevitably at the expense of understanding the irreconcilability of many of these methodologies and the need for independent critical perspectives. Therefore, we will question what kind of epistemologies implementation science is working from and seeking to contribute to and whose interests are ultimately being served from such an endeavour.

## Inception of a new field of study

The rationale for focusing on implementation emerges from concerns about clinical effectiveness and addressing the ‘implementation gap’ (Shojania and Grimshaw 2005). Two major UK reports informing this concern were *Waste Not, Want Not*, a chapter in the CMO’s Annual report (2005) which identified the will for clinical efficiency and *the Cooksey Report* (2006) which identified a ten-year gap between innovations and their implementation in practice. These reports set an agenda in motion to decrease the ‘implementation gap’^1^.

Accompanying these reports has been the impetus to create an implementation research agenda which coincided with the inception of the journal *Implementation Science* in 2005-06. Before 2005, implementation research existed in several areas in evidence-based healthcare research such as knowledge translation/mobilisation and quality and safety improvement. It was the journal *Implementation Science* that brought these disparate fields together and first paired the words ‘implementation’ and ‘science’ together. The first issue of the journal in 2006 contained an editorial setting out the scope and aims of the journal. The Journal would be a place where: “implementation research articles [which were] scattered across a wide range of journals, including clinical, public health, health services, and healthcare quality/safety journals” would be collated into one place to give authors the ability to focus more on: “contextual, developmental and supporting work that would […] enhance the likelihood of successful replication of an intervention” (Eccles and Mittman 2006).

*Implementation Science*, therefore, aims to cover established concerns but with broader aspirations of seeking to form a distinct formalised ‘science’. This agenda has been cemented by wider cross national political will in the UK and US for the project. Resources have been invested by the National Institute of Health in the US in order to set up the Clinical and Translational Science Awards in 2012, which supports a national consortium of sixty interdisciplinary research institutes working together on translational issues (Glasgow et al. 2012). In the UK, funding from the National Institute for Health Research supported the establishment of thirteen regional CLAHRCs (Collaborations for Leadership in Applied Health Research and Care) in 2008, which have focused on improving the effectiveness and efficiency of clinical care^2^. These organisations have increased the amount of attention on implementation research but do not answer how the field should be defined or conceptualised (or in whose interests).

In 2006, around the time of implementation science’s inception, the Chief Medical Officer for England^3^ called for an expert board to be assembled, headed by Martin Eccles, and to set an agenda for implementation research (Eccles et al. 2009). On the agenda was the need to focus on challenging existing uses of methodology and a higher regard for the use of theory. The emergence of implementation science onto the health agenda has signified a space for new questions and the re-engagement of old paradigms.

To address these agendas, the field has been expanding and draws on relevant approaches from a wide range of sources. The Diffusion of Innovations Systematic Review from Greenhalgh et al. (2004, 588) demonstrates how many distinctions there are when attempts are made to identify the research traditions relevant to implementation. The review identified thirteen research traditions and sixty-three theories of implementation spread over the social sciences and medicine, ranging from: sociology, anthropology, economics, politics, and marketing to psychology and epidemiology (Greenhalgh et al. 2005). To navigate these diverse fields, the review recommends a ‘meta narrative’ approach of melding all results into compatible recommendations.

Understandably, the attempt to consolidate these vast arrays of knowledge is highly political and has focused on how to reach consensus on the implications for melding the study of ‘evidences’ with anti-positive methodologies in the social sciences (H. T. O. Davies, Nutley, and Mannion 2000; Iedema R 2009). This is intensified by the fact that the field has never reached consensus on its own terminology. Many overlapping fields of study exist that do not have their relation to implementation science fully defined. For example, terms like: knowledge transfer, knowledge translation, knowledge mobilisation, improvement science or quality and safety are all used in applied health care research. Authors such as Davies, Nutley, and Walter (2008) exemplify the political nature of these debates when arguing how terminologies used in implementation science may not be compatible with the methodologies being brought into the field. For example, the concept of ‘knowledge’ or ‘science’ used in these titles may not reflect the ways in which ‘knowledge’ or ‘science’ are conceptualised as contextual, contested and contradictory in other social science methodologies.

The current state of the field is caught between tailoring research findings to applied health care whilst struggling to establish the field against other social sciences. We argue that this contradiction stems from implementation science as the result of a problem identified and formulated in the evidence-based movement and the political will to advance its agenda. The resulting debate, centred on making the implementation field coherent, means that less attention is paid to the effects implementation may have and in whose interests it is serving.

## Evidence-based practice’s interest in implementation

The use of implementation science as part of the drive for evidence-based-practice highlights some long-standing problematics, as questions of implementation science encroach on the study of cultural and societal processes that have long standing methodological contestations (Shlonsky and Mildon 2014). In the nineteenth century, the sociology foundered by Auguste Comte was envisioned as a positivist science. His belief in positivism was that, just as in the natural sciences, valid social knowledge can only be derived from verifiable data received from the senses. Before the century was out, figures like Heinrich Rickert and Wilhelm Dilthey had transformed the debate to argue that as society is human experience, it is qualitatively distinct from and therefore should not be subject to the same methods as the natural sciences. A long standing dialectic has run ever since (Fuller 2013, chap. 3). Oppositions to positivism have focused on how meaning and interpretation are integral to understanding human interactions. Protagonists of anti-positivist positions have argued that the norms, values and symbols of human relationships are subjective and defy quantification and that it is impossible to demarcate an interpretation of society as value free or free of biases (Henrik von Wright 1975). As a result, methodologies in the social sciences have fragmented to develop a range of approaches criticising positivistic assumptions. Many social scientists have moved away from positivism in favour of interpretivistic, reflexive or critical approaches such as phenomenology, critical theory, post-structuralism, ethnomethodology, symbolic interactionism and process-philosophy, most of which emphasise their irreconcilability with positivism. In response, implementation science (to be discussed) has offered a middle way to appease both sides of such debates with alternatives such as post-positivism, middle theory or critical realism. However, implementation research that emphasises consolidation of these methodologies still does so in the face of the lessons learned from these traditions and at the expense of fostering an independent locus for criticisms of evidence-based methodologies.

Similarly, medicine has traditionally been conceived as a balancing act between being a science and an art/craft/technique. Scientific method underpins medical procedures as closely as possible, but the application of medicine in practice requires the ‘art of medicine’ i.e. the skill/judgement of the practitioner to interpret and utilise effectively (Kelly and Moore 2012). In an attempt to improve efficiency, evidence-based medicine has focused on the direct observation of the effectiveness of medical treatments (over aiming to understand the underlying causes or ‘mechanisms’ of illnesses and treatments). This is done by classifying and prioritising evidence of effectiveness according to the extent to which results are verifiable (in the same sense as positivism). This has had complex implications on professional power and the standardisation of the ways medicine has been structured and organised (Timmermans 2005; Timmermans and Mauck 2005). Consequently many controversies still remain unresolved as to the extent evidence-based medicine can be spread. Some aspects of practicing medicine are not available to scientific method in the same ways as other ‘natural sciences’ (Stengers 2003). For example, how do such methods apply to highly discursive fields such as psychoanalysis or faith healing?

Irrespective, since its inception, the evidence-based approach has spread to founder broader movements of evidence-based practice and evidence-based policy (a good example of this is the Campbell Collaboration^4^). These developments raise the question of how evidence is informing medicine in the broadest sense including policy, practice and healthcare. In evidence-based medicine the dualism between medicine as a science or an art/craft/technique is not tackled head-on but subverted by the ‘hierarchy of evidence’ (Guyatt et al. 1995). This hierarchy prioritises positivistic ‘evidence’ such as systematic literature reviews and randomised control trials at the top and non-controlled or observational studies towards the bottom. The question remains, however, whether some sources of evidence are ever open to systematisation or synthesis, and the extent to which ‘evidences’ are open to interpretation. For example, implementing innovations may be a question of balancing evidences, resources and other factors; evidence in the clinic may be contingent or time specific; or evidence may have other ethical ramifications in its collection (Nutley, Powell, and Davies 2013). David Sackett, one of the founders of the evidence-based movement, famously stated that “Evidence-based medicine is not “cookbook” medicine” (Sackett et al. 1996, 71) in response to criticisms that a focus on ‘evidence’ obscures practitioner judgement, which at the time had the effect of reaffirming the aims and approach of evidence-based medicine. Subsequent models of the hierarchy of evidence have integrated professional expertise and patient values and preferences as crucial to the application of the hierarchy model (Sackett et al. 2000).

However, with the arrival of implementation science (and accompanying technologies), ways of organising evidence are pushed ever further^5^. Implementation is at the forefront of the systematisation of evidence in medical practice, and implementation science is a field envisioned to facilitate evidence-based medicine. So far, implementation science has fulfilled this purpose pragmatically. The field is being constructed around theoretical frameworks that aim to systematise the implementation of ‘evidence’ (see the frameworks demonstrated in Table 1). However, more and more concern is being raised by researchers working with qualitative data in the field, especially around variability and reaching consensus over best evidence and how criticisms are accounted for (exemplified in agencies such as “the Alliance for Useful Evidence”: Nutley, Powell, and Davies 2013). In these concerns, the strain on the paradigm of making research findings conform to being forms of ‘evidence’ can be seen to increase.

Implementation science achieves operationality through the recognition of the need for evidence beyond that which is quantifiable. The field of implementation science has become possible due to a change in trends as to what constitutes evidence in medicine. This is reflected in the MRC’s (2008) Framework for Developing and Evaluating Complex Interventions (the most current iteration of UK policy and guidance for practitioners planning health interventions), ^6^ which endorses the importance of qualitative methodologies, depending upon the research question. The recommendation to use both quantitative and qualitative methods signals the desire of implementation research to maintain a more sustained dialogue with the social sciences.

Of concern is the ways in which it is attempting to do so. Such dialogues seem to be solely on the level of judging the reconcilability of social science methodologies with evidence-based methodologies. The problem, however, is that many of these traditions are founded precisely on their irreconcilability with positivism and are more readily sceptical or critical of the boundaries of the evidence-based movement. Traditionally, such critical perspectives have served to counter evidence-based medicine independent from the resources, power structures and vested interests that evidence-based medicine may or may not serve (Boulton 2017). To what extent are they to be accounted for in implementation science? For example, how prominent are debates that question the extent to which implementation frameworks and guidelines may exacerbate issues such as the Taylorisation or bureaucratisation of a care service, the destabilisation of management/employee hegemonies, profiteering or best care in hard to define or highly individualised cases? While there is no shortage of critical academic debate on the subject of evidence-based medicine, the issue is how it is being serviced in frameworks and guidelines that are designed to be used independently in care settings by services practitioners. Implementation science’s current approach to evidence, epitomised by the MRC’s Guidance on Complex Interventions (2008), recognises the need to draw on qualitative methodologies but does so in the pragmatic instrumented style of quantitative methods more established in the evidence-based movement. The coining of terms in implementation science, such as ‘meta narrative’ or ‘evidence synthesis’, do nothing to allay the fears that all methodologies must be incorporated to fit one concise overriding implementation approach. The significance of these questions will grow in line with the growth of the implementation field to spread evidence based methodologies in to wider frontiers.

## Conceptualising implementation science

Implementation science emerges out of an agenda that seeks to address clinical effectiveness. As a result, much implementation research does not stem from attempting to place implementation into a wider cultural/historical framework; rather, it stems from a pragmatic paradigm aiming to improve healthcare practices and patient outcomes. As demonstrated, this has some important historic reasons; however, it masks wider reflections around how the field is being imagined and configured. As well as confronting the question, *how should we implement,* important questions can be raised by asking, *how is implementation being conceptualised* and *in whose interests it is being geared to serve.*

The formal study of implementation as a subject in its own right has had an existence independent from, although little referenced by, the areas of healthcare, organisation or policy it is now being applied to as a formalised science. The (now defunct) field of policy implementation research started in the 1970s and spanned thirty years, in which time the field sought (and failed) to establish a grand theory of implementation. The initial field was outlined in three initial studies by Derthick (1972), Bardach (1977) and most comprehensively Pressman and Wildavsky (1973), who proposed a general definition of implementation. From these discussions the field moved through two broad distinctions between authors who argued for an approach to implementation that was top down (i.e. from policy makers down to implementers) and authors who sought to demonstrate how implementation was also bottom up (i.e. from the street level to implementers to policy makers) (see Lipsky 1980; and Nakamura and Smallwood 1980).

Further generations of authors took elements of both and combined them into integrative and predictive models of implementation (see Hjern 1982; Sabatier 1986). For thirty years, policy implementation research sought to establish a general theory of implementation before the pursuit was abandoned in favour of studies that focused on describing implementation in specific programmes and enveloping the study of implementation more generally as part of the study of overall policy processes (deLeon and deLeon 2002; Conteh 2011). Just before the decline of policy implementation research in 1990, Fox (1990) observed that in the twenty years since Pressman and Wildavsky defined the field, implementation policy research had shadowed the historical developments of the social sciences (from positivism to phenomenology), which leaves us to question if implementation science is fated to repeat a similar revisionist history (also see Ingram 1990). This independent history of implementation has not been thoroughly developed or acknowledged in implementation science theory.

The only comparative history of the two disciplines (implementation policy research and implementation science) comes from Nilsen et al. (2013). Nilsen et al. acknowledges the warning that comes from the demise of implementation policy but downplays the dangers, concluding that implementation as conceived in implementation science is different to that of policy implementation research as implementation science has the more robust notion of evidence to fall back on. However, considering that Fox (not cited in Nilsen) pinpoints the problem with implementation policy research precisely with positivism (or in other words the search for evidence), the issue should be highlighted as needing more consideration in implementation science. The few other authors of histories about implementation science, such as Dearing and Kerk (2012)^7^, choose not to cover policy implementation research at all. This omission raises the question of how the history of such issues are being framed (or not) and whose interests such a framing is in.

Such histories demonstrate implementation science’s emergence from an evidence-based paradigm. These paradigms are mostly concerned with identifying evidence and making it inform practice and, as a result, under represent wider historical and cultural understandings of implementation as studied independently from evidence-based approaches. Rather than demonstrating implementation science as emerging from academic or research concerns, it highlights implementation as a large-scale political and economic manoeuvre and demonstrates how the focus of this endeavour has been the pragmatic coordination between relevant institutions and bodies. The effect of such manoeuvres put implementation in danger of being normative in its approach and obscures wider questions of in whose interest’s implementation is serving.

In principle, the implementation of research evidence into practice could be seen as relevant to a number of disciplines. However, the ways that different disciplines may frame the concept of implementation methodologically and epistemologically may be contradictory. The evidence-based movement’s interest in implementation is to create an operational model of implementation that can be readily used in practice (Davidoff 2011). This may be at odds with other epistemological perspectives that may be more reflexive on the concept of implementation or critical of its effects. Implementation science acknowledges that producing a model of implementation using evidence-based methodology exclusively is unattainable, hence the need to create a new field of implementation science to bridge different disciplines. Less acknowledged, however, is the how this quest for compatibility may result in dominance over outlying counter voices or concerns. To assess such concerns, the remainder of this article will focus on the ways the theory and practice of the field are being conceived.

## Implementation science in practice

A plethora of theoretical frameworks already exist in implementation science. The dominant mode of operation in the field is the production of normative frameworks conceptualising implementation. This theorising is presented as 'middle-range theory' (Davidoff et al. 2015). Middle-range theory is not intended to constitute a theory in itself but is an approach to the process of assembling theory from the social sciences. Implementation science uses middle-range theory to free the field from attempting to create overarching, universal explanations of the sociality it seeks to make interventions into. Instead, implementation science entails approaching theory as aligned to one social phenomenon without a universal application outside of the theories intended purpose.

This means that the field is geared around a set of frameworks that can fit together (see Table 1). To illustrate how this principle is adopted in framework design, an obvious example can be found in the Theoretical Domains Framework (TDF), which was developed by an ‘expert panel’ to consolidate existing theories of behaviour change into a concise set of recommendations (French et al. 2012). The TDF attempts to order thirty-three theories and 128 explanatory constructs of behaviour change techniques (a major component of implementation) into twelve specific domains (French et al. 2012). The approach suggested for users of the TDF by its advocates is to identify a theory as compiled in the list. The framework claims legitimacy by demonstrating how it is cumulative around other theories of implementation, as can be seen here:

The TDF is potentially compatible with a range of existing frameworks in the implementation literature. For example, Kitson et al. (2008) called for the integration of theoretical perspectives into the PARHIS framework. The TDF could be useful in elaborating some components of the 'diagnostic and evaluation' stage of PARIHS. Damschroader et al. proposed CFIR; there is potential for mapping the TDF domains on to constructs in this framework (in particular, within Outer Settings, Inner Settings, and Characteristics of Individuals). The advantage of such a process would be to provide access to a large evidence base from the behaviour-change literature that could be useful in CFIR-based research. (Francis, O’Connor, and Curran 2012, 7)

In this extract, a distinct field of study is being 'imagined' and defined with corresponding terminology. A set of key frameworks (TDF, PARIHS and CFIR) are identified as foundational and central to the field. There are many other frameworks designed for the field which are beyond the scope of this article, but these have been chosen here because of their prominence and the fact that they reference each other. These frameworks are promoted as cumulative, and users are encouraged to build upon them.

Frameworks in implementation science are designed to conceptualise an intervention to a specific aspect of implementation with the aim of acting as a procedural guide to aid implementers to make evidence informed interventions to practice.^8^ For example, PARIHS promotes action, TDF sorts organisational behaviour, CFIR standardises implementation, SQUIRE reports excellence and multiple others exist.

Implementation is conceptualised and broken down into a series of components, and then these components are assembled into a framework that represents how to operationalise interventions in practice. Each framework is designed to preside over an individual domain contributing to gaps in knowledge without overlapping with another. This can be demonstrated with CFIR, for example. CFIR is built upon the Diffusion of Innovations systematic review (Greenhalgh et al. 2004) and uses a snowball sample to compile further implementation theories into a framework that conceptualises the major domains studied in the field. In effect, CFIR is attempting to coordinate studies in the field, align them side by side, make different frameworks compatible and postulate the borders of the field. CFIR quotes PARIHS to demonstrate CFIR’s compliance with the field to uphold the principle that the successful implementation of evidence relies upon:

...professional consensus within a particular scientific community. [Implementation] stands for the entire constellation of beliefs, values, and techniques shared by members of that community... [and] need not specify the direction of relationships or identify critical hypotheses. (Damschroder et al. 2009, 3; Rycroft-Malone 2004, 298)*.*

In this way, the field of implementation science is framed as a coherent and singular mode of operation, shaped by evidence-based empiricism. These interventions are inventing, making visible and standardising the field. This opens up questions of how frameworks gain authority and the extent to which the building of frameworks is initiated as the result of professional projects. Aptly, the arrangement of theory flexibly accommodates new and old frameworks.

Conceptualising theory as a set of domains serves to frame implementation science as cumulative and aligned to the empirical sciences (in contrast to the social sciences). Working from the premise that implementation needs to be cumulative, the consensus has been to build a base that can give clear recommendations to practitioners.

However, the field is in danger of spreading a normative conception of implementation. By emphasising cumulativeness, implementation frameworks reflect assumptions of the same monist, positivistic methodology. Cumulative approaches arguing that evidence-based methodologies are designed to bring about the ‘closing the implementation gap’ and ‘effectiveness’ are ad hominem arguments. For example, the success of ‘closing the implementation gap’ or any increase in ‘effectiveness’ is judged by the presence of ‘good evidence’, and the definition of ‘good evidence’ is judged by its ability to ‘close the implementation gap’ or increase ‘effectiveness’. As a result, within CFIR (or other leaders in the field such as PARIHS, TDF) some questions are purposefully omitted; for example, what *is* implementation, evidence, or context? How are service hierarchies imagined and accounted for? How do ideal targets get decided? Who gets to judge the usefulness and adaptability of the theory? In whose interests do the outcomes ultimately serve? And are class or area disparities considered? Conceptualisations of implementation, evidence and context become black boxed and forgo the myriad of associated conceptions they can be broken down into. Taken to extremes and coupled with wider trends such as the digitisation of medicine, such frameworks threaten to reconfigure wide ranges of relationships, standards and established definitions of what constitutes care and medicine in specific settings. If the overall coherence of the field is overemphasised, it may result in the disparagement of other reflexive or critical engagements with healthcare.

Therefore, the use of middle-range theory in implementation science is alarming. In the years since Merton coined middle-range theory in 1949, science and technology studies (STS, the parent discipline Robert Merton helped to establish) have come to emphasise that theory should have no *a priori* assumptions regarding the use of epistemology and methodology (Zuiderent-Jerak et al. 2009; Zuiderent-Jerak 2007). Raymond Boudon (1991) noted that an important factor in operationalising middle-range theory is that competing theoretical frameworks must be conceived as allowing for contingency, contradictions, overlaps and not be absolute. In other words, middle-ranged theory should not be cumulative and fit together consistently. One theoretical lens is not correct in every circumstance but must be specified (Bogusz 2014; Geels 2007).

With a bias toward the evidence-based movement, as identified, questions can be asked about the purposes implementation science envisages in its use of middle-range theory. Implementation science is caught between appeasing the evidence-based movement whilst defending the field alongside other social sciences. For this reason, much attention in implementation science has been drawn to maintaining the concept of cumulativeness with that of middle-range theory by aligning it with critical realism to explore the extent theories can be ‘federated’ together. As we shall argue in the next section, this stance closes down a number of critical and reflexive stances in the field.

## Implementation and middle-range theory

Merton developed 'middle-range theory' in the social sciences to contrast with the scope of more general theories of society. As a student of Talcott Parsons, Merton saw at first hand the struggle to unify positivism and hermeneutics into a general unified theory of sociology. Merton’s scepticism about such a large undertaking motivated him to propose middle-range theory as a way to appease the impassable contradictions across the sciences by moderating the scope of social theory to focus on specific applications. Disciplines of the empirical sciences, such as physics, have a unifying method. In contrast, the social sciences form camps between positivistic, interpretivistic and critical approaches and fail to reach consensus on a unifying method or principle of society or human. As a result, the social sciences/humanities have developed a reflexivity in their use of method and representations of reality and, in general, have accepted the irreconcilability of methodologies and epistemiologies.

Interpretivist or critical methodologies of the social sciences contest singular, aggregated approaches on how organisations, innovations and improvements function in favour of specialising in elucidating nuances and problematics. This is in contrast to the evidence-based movement that implementation science is emerging from, which emphasises cumulation. Cumulative approaches seek to build on established knowledge, but in the process can obscure ulterior perspectives. Something like an implementation science would have already existed if the social sciences or humanities had been successful over the last century at offering a satisfactory general theory of society that could explain organisational change and the uptake of innovation (Griffiths 2003; Davies 2003).

Just a few of the methodological incompatibilities still being worked out in implementation science are positivism; middle-range theory which stems from, but radically alters, structural functionalism and hence forms a complex relationship with both positivism and interpretivism; critical realism, which has run parallel to the social sciences and also heavily modifies both positivist and interpretivist social sciences; and programme theory/logic modelling, which done unreflexively is implicitly positivist despite encouraging the use of interpretative and qualitative methodologies.

This highlights the reasons why implementation science has found such a use for middle-range theories, as it allows for inconsistencies between theories (Takian et al. 2012; Bate et al. 2014). Davidoff et al. (2015) frames implementation science as working along the lines of middle-range theory. However, it remains to be seen what the implications will be for such one-sided versions of the middle-ranged theory concept being adopted. For example, if middle-range theory is being used here to allow researchers to forgo contradictions to produce generalizable findings, does that allow for non-empirical (i.e. critical) findings? Some in the field have already reached a conclusion to draw more heavily on empiricist interpretations, visible chiefly through the work of Ray Pawson. He allies middle-range theory with critical realism to suggest that:

The plan is that researchers would use the same apparatus or model to pull together explanation in diverse substantive fields. A heavy editing of Merton’s closing summary (1968 p68) reveals the big idea: “middle-range theories consist of limited sets of assumptions from which specific hypotheses are logically derived and confirmed by empirical investigation… These theories do not remain separate but are consolidated into wider networks of theory… [that are] sufficiently abstract to deal with different spheres of social behaviour and social structure.” I now turn to ask why it has been relatively little practiced? Seldom does one read a paper promising a ‘federated, middle-ranged theory of…. (Pawson 2000, 289)

Drawing on Pawson’s work combining middle-range theory and programme theory authors like Carl May (May 2013; May and Finch 2009) envisage a general theory of implementation (linked to the NPT model generated in sociology see Table 1.3) by ‘federating’ existing middle-range theories. This contradicts much of the traditional thinking and usefulness of middle-range theory, as according to Merton and Boudon middle-range theories do not have to add up to one comprehensive theory nor are they necessarily hierarchical or cumulative. Therefore, a contradiction in the field exists concerning theory building, which begs the question as to why the aging (and some would say superseded) middle-ranged theory is so heavily emphasised in the face of decades of further developments in STS and of a whole host of other critical or reflexive methodologies? Given the youth of the field, the conviviality of such open and heady (‘middle-ranged’) dynamics may permit creative licence for a whole raft of professional projects to be created.

This is an important question as it concerns how knowledge about implementation is made authoritative and where authoritative knowledge originates; for example, from the top down from expert frameworks or from the bottom up from patients and those in direct contact with them (Ferlie et al. 2005; Dopson and Fitzgerald 2006). While it is unrealistic to imagine any field of study without contradictions, what needs further reflection in implementation science, however, is how the political impetus to create a discipline opens opportunities for many vested interests to take hold.

## Conclusion

This article has framed implementation science as part of a trend to widen the scope of the evidence-based movement to more areas of healthcare. Part of this process is to question what constitutes evidence and the values placed upon different types of evidence (see MRC complex interventions). Implementation science addresses the problem by drawing on wider accounts in the social sciences that are hard to accommodate with evidence-based methodologies. For this reason, importance has been placed upon theory building in implementation science, and this paper has questioned how the process is taking place within the field, what is omitted, and in whose interests it serves.

Much of the field is imagined through a concept of middle-range theory. Implementation frameworks draw on middle-ranged theory because it emphasises openness and flexibility in theory building. However, caution must be highlighted as to the perceived universality of ‘middle-ranged theories’ when conceived as cumulative or contributing to a ‘general theory’ of implementation. If, for example, healthcare findings must be made compatible with positivist methodologies, the space to incorporate incompatible or critical methodologies becomes limited. The conceptualisation and evaluation of the process of theory building in implementation science lacks reflection of the divide between empiricism/positivism, hermeneutic interpretivism and other critical methodologies.

As the impetus for founding a new field lies in the evidence-based movement (the implementation gap and clinical effectiveness) and the method to achieve these goals is to engage with wider disciplines and epistemologies, the emerging implementation science field should evaluate (and be reflexive and critical of) what this melding of epistemologies will imply for its practice and application in complex healthcare services. If implementation science gains ground traditionally occupied by the humanities and social sciences, it is at risk of spreading a normative positivist methodology, the dangers of which have long been documented in the same disciplines attempting to be drawn from. For this reason, caution should be placed upon the extent that theories of implementation can be generalised for use over many diverse services and settings.

Methodologies of the social sciences and humanities are recognised as productive and not merely descriptive. Therefore, their usage will influence the healthcare services they are applied to. Without independent criticism, cumulative understandings of ‘research evidence’ and the goals of ‘closing the implementation gap’ and ‘effectiveness’ –that evidence-based methodologies are designed to bring about—are self-fulfilling *ad hominem* arguments. For example, the success of ‘closing the implementation gap’ or any increase in ‘effectiveness’ is judged by the presence of ‘good evidence’, and the definition of ‘good evidence’ is judged by its ability to ‘close the implementation gap’ or increase ‘effectiveness’. Much emphasis is placed upon measurement in the field, but the danger is that implementation science theory has no perspective with which to interpret improvement or efficiency outside of a concept of evidence. Therefore, how can we fully assess the claims of proponents of implementation science, such as those presented at the beginning of this article, i.e.: “to improve the quality and effectiveness of health services and care”? Any interpretation of improvements either needs to be translated as ‘evidence’ or will be ignored. In this way implementation science is in danger of being self-serving, or politically serving the interests of those with a vested interest in the field.
